# Spatial physical activity patterns among primary school children living in neighbourhoods of varying socioeconomic status: a cross-sectional study using accelerometry and Global Positioning System

**DOI:** 10.1186/s12889-016-2954-8

**Published:** 2016-03-22

**Authors:** Rahel Bürgi, Laura Tomatis, Kurt Murer, Eling D. de Bruin

**Affiliations:** Department of Health Sciences and Technology, Institute of Human Movement Sciences and Sport, ETH Zurich, Zurich, Switzerland

**Keywords:** GPS, GIS, Accelerometer, Physical activity, Sedentary behaviour, Location, Neighbourhood socioeconomic status, Children

## Abstract

**Background:**

Neighbourhood socioeconomic status (SES) has been shown to be related to health status and overweight independent of individual SES. However, results about the association between neighbourhood SES and physical activity among children are ambiguous. Particularly, it is unknown how socioeconomic factors influence the spatial context of children’s moderate-vigorous physical activity (MVPA) and sedentary behaviour (SB). This study aimed to investigate by means of Global Positioning System (GPS) and accelerometry whether locations where children engage in MVPA and SB differ by neighbourhood SES.

**Methods:**

Participants included 83 children aged 7–9 from nine public schools located in a low- and high-SES area in Zurich, Switzerland. Children wore an accelerometer and GPS sensor for seven consecutive days. Time-matched accelerometer and GPS data was mapped with a geographic information system and each data point assigned to one of eight activity settings. The amount and proportion of MVPA and SB were calculated for every setting. To investigate differences between the two SES groups, multilevel analyses accounting for the hierarchical structure of the data were conducted.

**Results:**

Both SES groups achieved most minutes in MVPA at own school, on streets and at home and recorded the highest proportions of MVPA in recreational facilities, streets and other schools. The highest amounts and proportions of SB were found at home and own school. High-SES children accumulated significantly more minutes in MVPA and SB in parks, sport facilities, other schools and streets, while the low-SES group spent more time in both activities in other places. When taking the total time spent in a setting into account and using the proportion of MVPA or SB, the only differences between the two groups were found at other schools and outside, where the high-SES children showed a significantly higher activity level (*p*-values <0.001).

**Conclusions:**

Several differences in the spatial activity pattern between children from low- and high-SES neighbourhoods were found, independent of their individual SES. The findings seem to highlight the importance of providing safe streets and access to appropriate types of recreational facilities to reach recommended PA levels. Further policies to reduce SB within home and school environment are needed.

**Electronic supplementary material:**

The online version of this article (doi:10.1186/s12889-016-2954-8) contains supplementary material, which is available to authorized users.

## Background

The promotion of an active lifestyle among youth is crucial given that the benefits of regular physical activity (PA) are well acknowledged [1–3] and many health behaviours may track into adulthood [4, 5]. Moreover, physical inactivity was globally identified as one of the four leading risk factors for non-communicable diseases causing more than three million deaths per year [6]. Different studies investigating current levels of PA among children and adolescents in Europe reported that only a small percentage meets the recommended PA guidelines of 60 min of daily moderate-vigorous PA (MVPA) [7, 8]. Such activities require a high energy expenditure (≥3 METs) and result at least in raising the heart-beat and leaving the person feeling warm and slightly out of breath. In general, MVPA is seen as health-enhancing physical activity which is defined as any form of PA that benefits health and functional capacity [9]. Along with the alarming European situation, only 20 % of the boys and 11 % of the girls aged 11 years are adequately physically active in Switzerland [8].

In addition to the level of PA, sedentary behaviour (SB) has generated much interest among researchers in recent years. SB refers to behaviours that require a low level of energy expenditure (≤1.5 METs) and are mostly spent sitting [10]. Being sedentary is distinct from being physically inactive and, therefore, cannot be defined as the absence of health-enhancing PA [11]. There is growing evidence that SB can affect the health status in children and youth distinct and independent of PA levels [12]. Different studies demonstrated that SB is highly prevalent and children spend up to nine hours of their daily waking time sitting [12, 13]. As a result of this high prevalence of SB among youth, the promotion of an active lifestyle in combination with a decrease in levels of SB should be a fundamental component of public health. However, for the development of effective interventions, it is essential to have a clear understanding of children’s PA and SB patterns and which factors influence these behaviours [14].

One factor often thought to be an important correlate of PA and SB is socioeconomic status (SES) [13, 15], which is usually determined by individual factors such as educational attainment, household income or the occupation of a person [16]. Previous research reported that these factors have an important influence on health status and the prevalence of being overweight [17, 18]. The participation in leisure-time PA and the membership in sports clubs further seem to be positively associated with individual SES [19]. Different studies also demonstrated that children with a lower individual SES have more access to electronic media devices [20] and spend more time in SB such as TV viewing [21, 22]. Nevertheless, results about the association between individual SES and SB as well as PA still are ambiguous and little is known how socioeconomic factors influence the daily patterns of these behaviours in school-aged children [15, 21, 23, 24]. A previous review by Beenackers and colleagues [25] further showed that the influence of socioeconomic inequalities among adults differed greatly by domain of PA, although there was no clear difference in total PA. This result may be an indicator for the importance of focusing on specific domains and settings and indicates that the choice of total PA or SB as outcome measure may not be suitable when investigating associations between PA or SB and SES. However, it is unclear at present if similar patterns already exist in childhood and whether children with varying socioeconomic backgrounds use different spaces to be physically active or inactive.

Along with the increased use of ecological models [26], researchers have recently begun to focus more on the environment in which people live. Therefore, also the neighbourhood has been recognized as an important correlate of PA and has become an increasing focus of research [27, 28]. Different studies reported that neighbourhood SES, commonly measured using area-level variables such as percent unemployed or median household income [16], may influence resident’s PA independent of individual SES [27, 28]. In terms of SB, few studies investigated the influence of the neighbourhood environment on time spent in SB. Although initial findings point to a stronger influence of proximal factors such as home and family environment [29, 30], there is some evidence that children living in socioeconomically disadvantaged neighbourhoods are more likely to spend time in sedentary activities [31, 32]. However, most studies investigating the association between neighbourhood and SB in children used subjective self- or parent-reported TV or screen time, which is a very poor indicator of total time spent in SB [33]. Moreover, an identified research gap which needs to be addressed is the lack of studies investigating associations of active travel to destinations other than school with diverse sedentary activities beyond simply screen-based activities [34].

Increasing numbers of investigators have recently used Global Positioning System (GPS) devices along with accelerometers to identify spatial behaviours and how people use their surrounding built environment for PA [35]. With this technology, it is possible to objectively assess the amount of PA and SB and the location where these behaviours take place [36]. Several studies concluded that the simultaneous use of accelerometry and GPS provides reliable and accurate measurement of PA and its spatial context [37, 38]. Thus, the combination of accelerometry and GPS seems to be a promising method to gain further insights into differences in spatial PA and SB patterns among children from neighbourhoods with varying SES.

To conclude, only few studies investigated patterns of both PA and SB in different settings and domains by means of objective accelerometer-based measurements and among children from different neighbourhood SES. Understanding the influence of the social environment in which children live and how they use this environment to be physically active or inactive is an important step for future interventions [28]. Particularly, to guide interventions that target on improving local environments and supporting the design of outdoor spaces, especially in more deprived areas, insights into patterns of PA and SB within different locations among children from neighbourhoods with different SES are needed. Using the combination of GPS and accelerometry, the current study aimed to identify locations where children attending second grade spend time in MVPA and SB. In particular, the goal of our study was to investigate if there are any differences in the spatial PA and SB pattern between children from neighbourhoods with varying SES independent of their individual SES.

## Methods

### Setting

The current cross-sectional study was conducted in the municipality of Zurich between February and June 2014. Zurich is the largest city in Switzerland with a population of over 400’000 residents and is divided into seven school districts that differ greatly with respect to socioeconomic and environmental factors. To investigate differences in the spatial activity behaviour between children with different neighbourhood SES, we chose a convenience sample of second grade children from two districts that differ from each other within their neighbourhood socioeconomic standing. The standing was obtained from the Social Index (SI), which is a measure that quantifies the educational need for support of a school district or community. The SI is annually calculated based on three demographic characteristics, namely the proportion of foreigners, the social assistance rate and the proportion of low-income taxpayers [39].

### Participant recruitment

After approval of the study by the School and Sports Department of the City of Zurich, all teachers from a public school in one of the two districts and in charge of a second grade class were informed about the study in February 2014 by an information letter and invited to take part. If a teacher agreed to participate with his class, a trained study team consisting of the first author [RB] and a student research assistant visited the class at the end of March 2014 during a school hour and briefed the teacher and children about the study. During this visit, all children were provided with an information letter and an entry form for their parents, as participation was voluntary for every pupil in a participating class. The ability to engage in usual everyday PA and the absence of any severe disabilities were the only inclusion criteria. Parental written informed consent was obtained. The study was approved by the ethics committee of the ETH Zurich (EK 2013-N-66).

### Data collection and measures

During May and June 2014, all participating classes were visited again by the study team during a regular school hour one day before the start of the measurement. It was taken into consideration that measurements take place within a normal school week without irregular days off from school or school excursions. During this second visit, the study team fitted each registered child with an elastic belt equipped with a tri-axial accelerometer (GT3X, Actigraph, Pensacola, FL, USA) to measure PA and a GPS receiver (BT-Q1000XT, QStarz, Taipei, Taiwan) to record the geographical location (Circular Error Probability CEP (50 %) <3 m). The study team configured the devices for each child in advance setting both devices to record at ten second intervals with their internal clocks synchronized. This short sampling interval was used to accurately capture children’s PA pattern, which is characterized by short, intermittent bursts of activity [40]. Previous studies showed that both the accelerometer and the GPS device have acceptable accuracy for use in larger populations studies with a data collection period of seven or more days [41, 42]. Schipperijn and colleagues [41] previously demonstrated that 79 % of the data fell within ten meters of the expected location with the GPS device used in our study. All registered children were provided with a detailed verbal and written instruction on belt wear and further tasks to be done during the measurement week. They were asked to wear the belt around the waist from waking time to bedtime on seven consecutive days starting the next day. Each child was given a charger for the GPS device and was instructed how to charge the device during night when asleep. Children further received a small diary and a parental questionnaire. In the diary, children had to report throughout the week times they woke up and went to bed and times and reasons when monitors were not worn. The parental questionnaire served to assess age, sex, home address and sports club membership. From the same instrument, nationality, language spoken at home, parental education and parental income were obtained to determine factors of children’s individual SES. Nationality was defined as the number of parents with a Swiss nationality and subdivided into three categories (e.g. none, one or two of the parents with Swiss nationality). Finally, the study team measured children’s height and weight using a stadiometer (seca 213, Seca AG, Hamburg, Germany) and a digital scale (Beurer GS 12, Beurer GmbH, Ulm, Germany) with participants wearing light indoor clothing and shoes removed. For the whole measurement period, daily meteorological data such as mean temperatures (in °C), sum of precipitation (in mm), and the sum of sunshine duration (in min) were provided by MeteoSwiss [43]. Children were asked to return all instruments to school one day after the last day of measurement, where the study team collected everything.

### Data processing

Each participant’s GPS and accelerometer data was manually reviewed to ensure that both files contained adequate data. Given that cut-points for different activity levels are based on the vertical axis, only accelerometer data from this axis was used for analysis. The GPS and accelerometer data were matched by date and time using existing software (Actilife 6.5.2, Actigraph, Pensacola, FL, USA), which produced for each recorded GPS point a measure of activity and location. The processing of the matched data was then performed using MATLAB R2012a (MathWorks, Massachusetts, USA) and R 3.1.2 (R Development Core Team, Vienna, Austria). Intervals with >60 min of consecutive zero activity counts were classified as non-wear time and excluded from analysis [44]. Activity records >5461 counts per 10 s were identified as outliers [45] and replaced with the mean of the previous and the following value. Based on age-appropriate cut-points [46, 47], each data point was then classified as sedentary (<101 counts per minute (CPM)), light (101-2295 CPM), moderate (2296-4011 CPM) or vigorous (≥4012 CPM) activity. Furthermore, the data was processed by visual observation as well as automatic identification of invalid GPS data points using extreme changes in distance and invalid values of altitude and removing them from the data.

### Activity settings

**Fig. 1:**
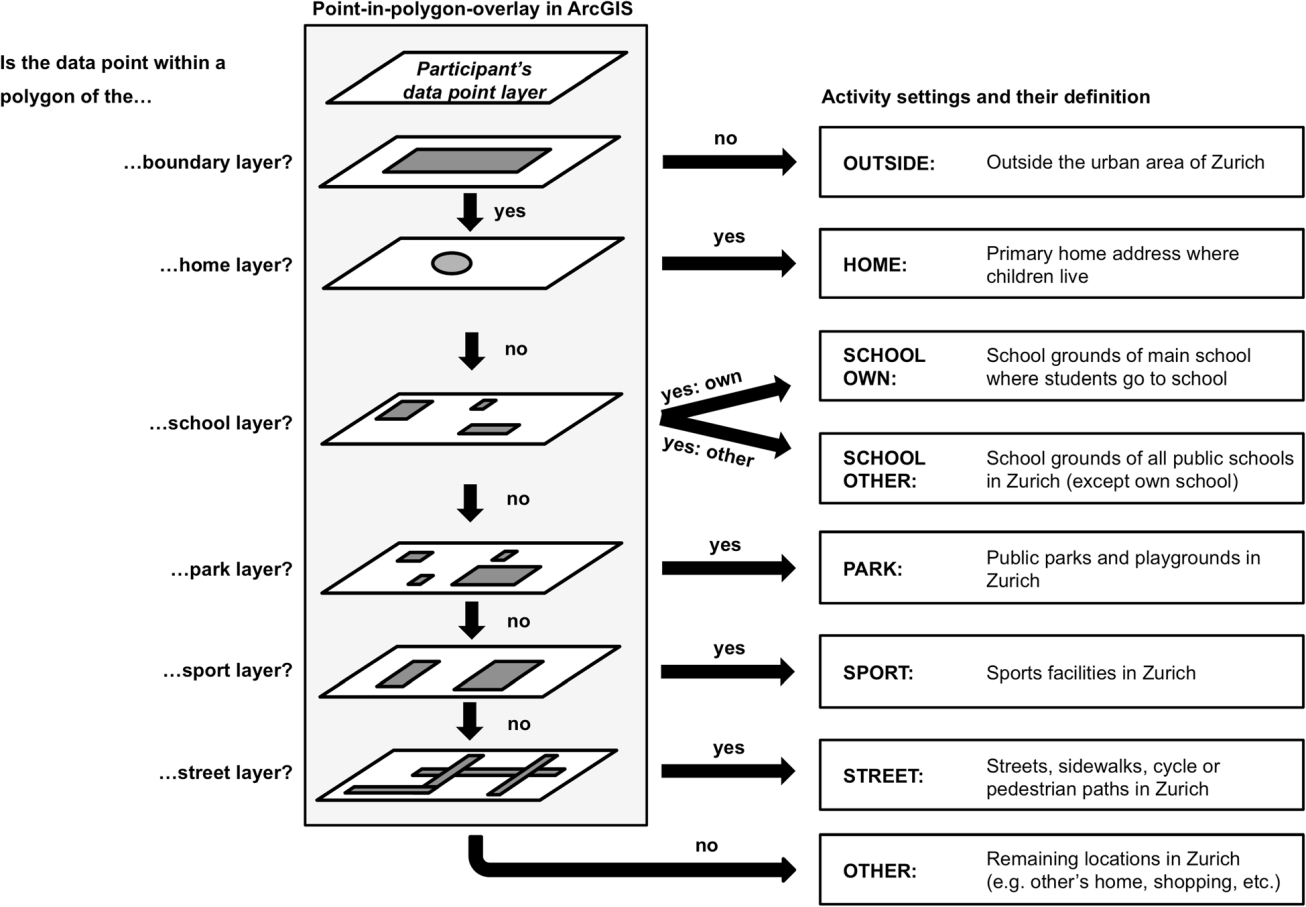
Definition of activity settings and overview of the assignment process. Legend: Using the point-in-polygon overlay in ArcGIS, each participant’s data point was assigned to one of the eight activity settings. This assignment process was conducted in a hierarchical order using six location layers. Each layer was created using further geospatial data provided by the Office for Geomatics and Surveying of the City of Zurich: By means of land use data, we digitalized the urban area of Zurich within a new layer *boundary*. The assessed home addresses from all participants were geocoded using Google Maps and plotted on the layer *home*. Within this layer, we created a 30 m Euclidian buffer around each geocoded home point to define home polygons. By using the points-of-interest file containing the location of all schools in Zurich and with the help of register data and satellite imagery, we manually digitalized all school grounds of any public school in Zurich and summarized them in the layer *school*. To take the measurement error of the GPS devices into account, we drew a ten meter buffer around each school polygon. The analogous procedure was carried out to construct a polygon layer containing public parks and playgrounds (*park*), and one layer containing sports facilities (*sport*). To create the layer *street*, we used land use data and selected all surfaces such as streets, sidewalks, cycle or pedestrian paths and drew a ten meter buffer around each polygon

The location-based categorisation of the matched data points was conducted in ArcGIS 10 (ESRI, Redlands, CA, USA). Each participant’s data points were imported into ArcGIS and plotted on a separate point layer. To assign each data point to a location, we chose to define the eight activity settings presented in Fig. 1, which are based on similar studies in this field of research [36, 48, 49] and the ability to clearly assign each point to a location within ArcGIS. The assignment process was conducted in a hierarchical order using the point-in-polygon overlay, in which each participant’s data layer was overlaid on six different polygon layers to determine which data points are contained within which polygons. The creation of these six polygon layers was done using further geospatial data (land-use data, register data, points-of-interest and satellite imagery) provided by the Office for Geomatics and Surveying of the City of Zurich and is described in Fig. 1. To take the measurement error of the GPS device into account, we drew a buffer zone of ten meters around the polygons of the layers school, park, sport and street [38, 41, 50].

### Data analysis

Only matched wear time data was used for analysis. To best capture all locations where children spent time in SB and PA, we required that they provide at least one day with nine full hours of matched data to be included in the analysis [49].

Statistical analyses were performed using R. To describe the general characteristics of the study population, we used descriptive statistics to calculate frequency distributions (number (n) and proportion (%)) for categorical data, mean and standard deviation (SD) for normally distributed variables, or median and interquartile ranges (IQR) for non-normally distributed data. To test differences between the districts in general and socioeconomic characteristics, univariate analysis was performed using *χ*^2^-test, *t*-test or Mann–Whitney-*U*-test.

As outcome measures, time in MVPA and SB per week (in minutes) was calculated for each setting and participant. Moreover, the proportion of time spent in MVPA and SB (in %) out of the total time spent in each setting was calculated. Median and IQR were used to present all outcome measures as these variables were not normally distributed. For the outcome variables, we further used multilevel analyses based on individual scores across days and locations. To improve the quality of data and models, we only included daily data from a specific location if the participant spent ≥2 min in this location on this day. Models were transformed by log transformation to fulfil the model assumptions and individuals and classes were included as random effects to account for the hierarchical structure of the data [51]. To adjust for individual differences, total wear time, week day, mean temperature, sum of precipitation, sex, BMI and sports club membership were included as potential confounders, as these parameters may have an influence on children’s activity pattern [52–54]. By separately adding the four ordinal variables language spoken at home, highest parental education, parental income and nationality to the model, we further adjusted for individual SES in order to assess the influence of neighbourhood SES on PA and SB independent of individual socioeconomic characteristics. A backward elimination algorithm with Akaike’s information criterion (AIC) as goodness-of-fit measure was applied to test the contribution of the entered predictors. The final models for the four outcome measures were included as Additional file 1. We then used a single-step-method to calculate contrasts between the high- and the low-SES district in every activity setting in order to investigate any differences between the two districts. Differences between the districts were provided by the transformed and adjusted *p*-values, which were calculated from F-test based on Sattethwaite’s or Kenward-Roger approximation. The study report following the Strenghtening the Reporting of Observational Studies in Epidemiology (STROBE) Statement (Additional file 2).

## Results

### General and socioeconomic characteristics

A total of 86 children from nine different classes provided consent and wore a measurement belt during one week. One participant had to be excluded due to illness and the associated abnormal PA pattern during the measurement week. One participant lost both devices and another one returned a malfunctioning accelerometer.

Thus, the final study population consisted of 83 children, including 38 children from the low-SES district, and 45 from the high-SES district. Overall, slightly more boys (51.8 %) than girls (48.2 %) took part in the study. As presented in Table 1, participants wore the devices during a daily median of 12.6 h at a median of seven days. During this time, they reached a median of five valid days out of seven possible days. After removing a total of 64,521 invalid GPS points during the data cleaning (3.3 % of the total matched wear time data), the GPS location was available for a median of 75.8 % of the total accelerometer wear time data. No significant differences were found between children with low or high neighbourhood SES in relation to anthropometric or wear time data (*p*-values >0.05).

**Table 1:** General and socioeconomic characteristics of the study population, in total and by district

		Total (*n* = 83)	Low-SES (*n* = 38)	High-SES (*n* = 45)	*p* - value
Age, mean (SD)	years	8.5 (0.3)	8.5 (0.3)	8.5 (0.3)	0.603**
Sex, n (%)	boys	43 (51.8)	23 (60.5)	20 (44.4)	0.107*
	girls	40 (48.2)	15 (39.5)	25 (55.6)	
Body height, mean (SD)	cm	133.4 (5.6)	134.2 (6.1)	132.6 (5.1)	0.196**
Body weight, median (IQR)	kg	28.6 (25.6 - 32.5)	30.1 (25.9 - 34.2))	27.2 (25.5 - 30.6)	0.088***
BMI, median (IQR)	kg/m^-2^	16.1 (15.1 - 17.4)	16.5 (15.3 - 18.1)	15.7 (15.0 - 17.1)	0.131***
Daily wear time, median (IQR)	hours	12.6 (11.8 - 13.3)	12.6 (12.1 - 13.4)	12.7 (11.5 - 13.3)	0.672***
Daily time of combined data, median (IQR)	hours	9.4 (7.9 - 10.3)	9.3 (7.8 - 10.3)	9.5 (8.1 - 10.4)	0.315***
Availability of GPS data, median (IQR)	%	75.8 (66.9 - 83.9)	73.8 (63.4 - 80.6)	78.4 (68.3 - 84.7)	0.113***
Wear days (daily wear time ≥1 h), median (IQR)	number	7.0 (7.0 - 7.0)	7.0 (7.0 - 7.0)	7.0 (7.0 - 7.0)	0.243***
Valid days (daily combined data ≥9 h, median (IQR)	number	5.0 (3.0 - 6.0)	4.5 (2.8 - 6.0)	5.0 (3.0 - 6.0)	0.823***
Language spoken at home, n (%)	German	48 (57.8)	13 (34.2)	35 (77.8)	<0.001*
	German and other	22 (26.5)	16 (42.1)	6 (13.3)	
	other	12 (14.5)	9 (23.7)	3 (6.7)	
	n/a	1 (1.2)	0 (0.0)	1 (2.2)	
Nationality, n (%)	Swiss	41 (49.4)	13 (34.2)	28 (62.2)	0.030*
	double citizen	25 (30.1)	15 (39.5)	10 (22.2)	
	non-Swiss	16 (19.3)	10 (26.3)	6 (13.3)	
	n/a	1 (1.2)	0 (0.0)	1 (2.2)	
Parental monthly income, n (%)	≤ CHF 4000	5 (6.0)	4 (10.5)	1 (2.2)	0.026*
	CHF 4001 – 6000	13 (15.7)	8 (21.1)	5 (11.1)	
	CHF 6001 – 8000	12 (14.5)	7 (18.4)	5 (11.1)	
	CHF 8001 – 12’000	22 (26.5)	10 (26.3)	12 (26.7)	
	≥ CHF 12’000	20 (24.1)	3 (7.9)	17 (37.8)	
	n/a	11 (13.3)	6 (15.8)	5 (11.1)	
Highest parental education, n (%)	compulsory/basic	4 (4.8)	4 (10.5)	0 (0.0)	<0.001*
	upper secondary	20 (24.1)	18 (47.4)	2 (4.4)	
	tertiary/higher	58 (69.9)	16 (42.1)	42 (93.3)	
	n/a	1 (1.2)	0 (0.0)	1 (2.2)	
Sports club membership, n (%)	yes	43 (51.8)	18 (47.4)	25 (55.6)	0.576*
	no	39 (47.0)	20 (52.6))	19 (42.2)	
	n/a	1 (1.2)	0 (0.0)	1 (2.2)	

Bold: significant difference at *p* < 0.05

*χ2-test; **t-test; ***Mann–Whitney-*U*-test

*IQR* interquartile range, *SD* standard deviation, *BMI* body mass index

In contrast, individual SES characteristics differed significantly by district (Table 1). Children from the high-SES district tended to rather speak German at home (*p* < 0.001), were more often of Swiss nationality (*p* = 0.03), and had more often parents with a high income (*p* = 0.026) and a tertiary education (*p* < 0.001).

### Moderate-vigorous physical activity

Table 2 provides the results on the PA level within the eight settings for the total population and separated by district. During the measurement week, children spent a median time of 416.3 min (IQR: 316.7–546.8) in MVPA. Irrespective of neighbourhood SES, children recorded most of their MVPA at own school (30.8 %), on streets (21.4 %) and at home (15.2 %). Although this order was identical in both districts, the high-SES group achieved significantly more minutes in MVPA in the street environment than the low-SES group (*p* < 0.001). Moreover, high-SES children recorded significantly more MVPA-minutes at other schools, in parks, sport facilities and outside (*p*-values <0.001), while low-SES children accumulated more minutes in MVPA in other locations (*p* = 0.024).

**Table 2:** Amount and proportion of time in MVPA across the eight settings, in total and by district

		Total		Low-SES		High-SES		
		n	Median (IQR)	n	Median (IQR)	n	Median (IQR)	*p*-value*
Amount of MVPA (in minutes)	Home	83	57.3 (32.2 - 91.8)	38	66.3 (38.7 - 105.3)	45	47.2 (31.5 - 76.7)	0.806
	Own school	83	121.5 (86.2 - 184.3)	38	125.2 (90.7 - 189.2)	45	118.3 (85.3 - 163.2)	0.160
	Other school	83	13.0 (3.3 - 28.2)	38	10.8 (4.8 - 22.0)	45	16.2 (3.2 - 33.2)	**<0.001**
	Park	83	9.3 (1.5 - 29.5)	38	3.3 (0.5 - 12.8)	45	18.2 (5.3 - 48.7)	**<0.001**
	Sport	83	4.3 (0.3 - 21.3)	38	1.1 (0.3 - 22.5)	45	6.7 (0.3 - 20.3)	**<0.001**
	Street	83	90.5 (56.0 - 127.0)	38	69.3 (42.7 - 111.2)	45	102.8 (72.0 - 135.7)	**<0.001**
	Other	83	42.5 (24.7 - 78.7)	38	56.4 (30.5 - 93.5)	45	35.2 (20.2 - 64.8)	**0.024**
	Outside	83	3.3 (0.0 - 19.5)	38	0.1 (0.0 - 10.5)	45	5.5 (0.0 - 30.0)	**<0.001**
Proportion of time in MVPA (in %)	Home	83	6.3 (4.8 - 9.3)	38	6.9 (5.1 - 9.3)	45	5.9 (4.7 - 8.5)	0.166
	Own school	83	10.0 (8.1 - 13.4)	38	10.1 (8.1 - 13.4)	45	10.0 (8.2 - 13.1)	0.092
	Other school	83	15.4 (7.1 - 23.8)	38	10.4 (6.4 - 19.4)	45	17.8 (10.7 - 25.0)	**<0.001**
	Park	79	17.3 (7.2 - 25.8)	35	16.5 (7.0 - 25.8)	44	18.2 (10.7 - 24.8)	0.601
	Sport	78	15.4 (5.9 - 33.1)	35	16.7 (5.4 - 29.6)	43	14.2 (6.4 - 36.4)	0.493
	Street	83	15.7 (11.7 - 19.7)	38	15.2 (10.5 - 18.3)	45	15.9 (12.9 - 19.7)	0.328
	Other	83	11.1 (7.8 - 15.6)	38	12.6 (7.4 - 16.1)	45	10.6 (8.6 - 12.8)	0.111
	Outside	51	8.4 (3.2 - 16.1)	22	4.5 (1.7 - 11.3)	29	11.7 (5.6 - 17.5)	**<0.001**

Bold: significant difference at *p* < 0.05

*p-values are calculated from F-test based on Sattethwaite’s or Kenward-Roger approximation

*IQR* interquartile range, *MVPA* moderate to vigorous physical activity

Taking the total time spent in a setting into account, the overall median proportion of time children spent in MVPA was 11.5 % (IQR: 9.0–13.3). As seen in Table 2, the low-SES group showed the highest proportion of time spent in MVPA in sports facilities (16.7 %), parks (16.5 %) and on streets (15.2 %), whereas high-SES children were most active in parks (18.2 %), at other schools (17.8 %) and on streets (15.9 %). Significant differences between the two districts were only found at other school and outside the city of Zurich, where the high-SES group showed a higher activity level (*p-*values <0.001). Furthermore, a non-significant trend was observed at own school (low-SES group: +0.1 %, *p* = 0.092).

### Sedentary behaviour

The children spent a median time of 2013.7 min per week (IQR: 1578.3–2386.5) in SB. Table 3 shows the weekly minutes of SB within the eight settings for the whole study population and separated by district. Across both groups, the majority of SB was accumulated at home and own school. Low-SES children compared to high-SES children recorded significantly less time in SB in parks (6.0 versus 47.7 min, *p* < 0.001), sports facilities (7.1 versus 10.2 min, *p* < 0.001) and on streets (193.9 versus 304.3 min, *p* = 0.015), while high-SES children spent significantly less time in SB in other places (198.3 versus 216.0 min, *p* = 0.011). Moreover, a trend of a difference between the two districts was detected at other schools (high-SES group: +0.9 min, *p* = 0.061).

**Table 3:** Amount and proportion of time in SB across the eight settings, in total and by district

		Total		Low-SES		High-SES		
		n	Median (IQR)	n	Median (IQR)	n	Median (IQR)	*p*-value*
Amount of SB (in minutes)	Home	83	529.7 (255.0 - 798.5)	38	538.3 (333.0 - 683.3)	45	470.3 (226.8 - 846.5)	0.776
	Own school	83	597.7 (509.0 - 731.7)	38	584.1 (477.2 - 724.7)	45	600.2 (533.3 - 749.2)	0.358
	Other school	83	46.7 (4.7 - 87.3)	38	45.8 (25.2 - 82.3)	45	46.7 (2.0 - 89.2)	0.061
	Park	83	15.7 (1.7 - 57.8)	38	6.0 (0.0 - 24.3)	45	47.7 (8.7 - 76.8)	**<0.001**
	Sport	83	8.5 (0.0 - 52.0)	38	7.1 (0.0 - 46.2)	45	10.2 (0.0 - 70.0)	**<0.001**
	Street	83	234.5 (173.3 - 378.2)	38	193.9 (123.2 - 313.5)	45	304.3 (207.5 - 397.0)	**0.015**
	Other	83	206.5 (130.5 - 304.2)	38	216.0 (131.2 - 342.5)	45	198.3 (130.5 - 287.5)	**0.011**
	Outside	83	26.5 (0.0 - 129.8)	38	16.2 (0.0 - 117.8)	45	34.3 (0.0 - 147.3)	0.901
Proportion of time in SB (in %)	Home	83	60.0 (52.4 - 64.5)	38	59.1 (53.3 - 63.9)	45	61.4 (51.5 - 65.7)	0.775
	Own school	83	51.7 (47.8 - 56.6)	38	50.7 (46.1 - 54.7)	45	52.8 (49.4 - 57.4)	0.664
	Other school	81	41.2 (30.8 - 55.1)	38	48.4 (30.8 - 64.0)	43	38.0 (30.0 - 50.6)	**<0.001**
	Park	78	37.6 (25.9 - 52.2)	34	30.9 (18.5 - 55.7)	44	40.2 (28.0 - 51.2)	0.609
	Sport	75	42.5 (21.3 - 62.4)	34	40.6 (21.3 - 56.8)	41	45.5 (22.2 - 69.4)	0.499
	Street	83	46.0 (39.9 - 49.7)	38	45.2 (40.6 - 50.1)	45	46.0 (39.1 - 49.6)	0.975
	Other	83	50.3 (43.4 - 58.0)	38	49.2 (42.8 - 55.9)	45	51.7 (45.7 - 58.8)	0.204
	Outside	51	52.5 (42.0 - 68.2)	22	61.9 (48.9 - 75.2)	29	48.3 (42.0 - 61.8)	**<0.001**

Bold: significant difference at *p* < 0.05

*p-values are calculated from F-test based on Sattethwaite’s or Kenward-Roger approximation

*IQR* interquartile range, *SB* sedentary behaviour

Overall, children recorded 52.2 % (IQR: 48.4–55.7) of their total wear time in SB during the measurement week. Outside (61.9 %), home (59.1 %) and own school (50.7 %) had the highest proportions of time spent in SB in the low-SES group (Table 3). The highest proportions among children living in a high-SES neighbourhood were observed at home (61.4 %), at own school (52.8 %) and in other places (51.7 %). However, we only found a significant difference between the two districts with high-SES children reporting a lower percentage of time spent in SB at other schools (-10.4 %, *p* < 0.001) and outside (-13.6 %, *p* < 0.001).

## Discussion

To the best of our knowledge this is the first study that objectively assessed the spatial context of MVPA and SB by means of GPS and accelerometry in primary school children living in neighbourhoods with varying SES. Our findings show that children from both districts achieved most of their minutes in MVPA at own school, on streets, at home, and in SB at home and own school, respectively. Streets, recreational facilities such as parks and sports facilities and other schools were highly conducive for MVPA among all children, whereas the likelihood of being sedentary was highest at home and own school. The high-SES group compared to the low-SES group accumulated more minutes in both MVPA and SB at other schools, in recreational facilities and on streets, while the opposite was true for other places. However, when taking the total time spent in a setting into account and focusing on the proportion of time spent in MVPA or SB, differences between districts were only found at other schools and outside the city, where high-SES children showed a significantly higher activity level.

Children from both neighbourhoods accumulated on average most of their health-enhancing PA on school grounds, streets and within the home environment. The importance of these settings in regard to the accumulation of daily MVPA was already observed by different studies [36, 48, 55] and can mainly be explained by the high use of these settings during the week. Overall, children recorded 32 % of their waking time at own school, 25 % at home and 16 % on streets, which is somewhat congruent with the accumulated time in MVPA. When taking into account the time spent in a setting and using the proportion of MVPA instead of the absolute amount, other schools and outdoor spaces such as parks, playgrounds, sports facilities and streets were found to be highly conducive for MVPA in both the high- and low-SES group. Accordingly, a recent study found that the proportion of time spent in MVPA was particularly high in active transport, playgrounds, sports facilities and urban green space [49]. It is well established that green space is highly supportive for MVPA, although the use of these settings as an absolute measure of time is low [49, 55, 56]. The high use of streets may be an indicator for active transportation or informal play and underlines the importance of these streets-based activities to reach recommended levels of PA [49, 57].

The current study further supports the finding that home and school environment is associated with generally low levels of recorded PA when using the relative amount of MVPA [56, 58]. Irrespective of neighbourhood SES, our participants recorded the greatest amount of SB at home and own school, where they spent more than 50 % of the time in SB. TV viewing and homework are two common activities that may account for the great portion of SB at home [59]. Oreskovic and colleagues [56] reported that indoor spaces are less conducive for PA than outdoor spaces. The high amount of SB recorded in the street setting among both districts could partly be explained by motorized transport. Motorized transport was found to be amongst the five most common sedentary activities in a sample of Scottish children [59].

In addition to the similarities between the two groups of children living in neighbourhoods with varying SES, we were also able to observe several differences in their spatial activity behaviour. Children from the high-SES neighbourhood accumulated significantly more SB as well as MVPA within parks and sport facilities. Given that both groups showed a similar proportion of time spent in PA and SB when taking into account the total time spent in these settings, this difference may rather be explained by a more frequent use than by a different behaviour within the settings. This assumption is partly supported by a higher density of parks in the high-SES district (about 30 %), which resulted in a four-fold increase in weekly dwell time (+1.5 h) among children living in the high-SES neighbourhood. Accordingly, this result confirms recent studies that highlighted the importance of proximity and access to parks and green space for meeting recommended levels of PA [60, 61]. In contrast, the more frequent use of sport facilities by the high-SES children cannot be explained by a higher density of sport facilities as this density was much higher in the low-SES district (about 100 %). Jones and colleagues [62] already found that residents from more deprived areas were less likely to use green space such as parks and sport facilities, although the accessibility was generally better. They concluded that also perceived access, problems with safety and the quality of the green space may play an important role in the use of these settings. Therefore, future studies and interventions have to address the individual needs of residents among different neighbourhoods to provide the appropriate infrastructure [62]. Previous studies further found that low-SES children engaged in more unstructured activities within their near neighbourhood, while high-SES children were more often encouraged by their parents through co-participation or logistical and financial support and, therefore, spent more time in commercial PA facilities or were engaged in sports club and organised activities [19, 52, 63]. Although we were not able to find a significantly different number of sports club memberships between the two districts, high-SES children were more likely to be a member of two or more sports clubs (18 % versus 5 %), while low-SES children predominantly reported to be a member of only one sports club (42 % versus 38 %).

The significantly higher amount of MVPA and SB accumulated by children from the high-SES neighbourhood within the street setting can also be explained by a different dwell time. This higher use of the street environment by the high-SES children may partly be attributed to differences in the settlement structure across the two districts. The high-SES children mostly lived in detached single or multi-family houses situated directly on a traffic-calmed street, whereas children living in the low-SES neighbourhood mainly resided in housing estates consisting of several apartment blocks with enclosed gardens equipped with playgrounds and playing fields. Therefore, it is likely that high-SES children more often used the street environment for informal play and traveling to a friend’s house or a public playground or park. In contrast, the low-SES group accumulated more minutes in both MVPA and SB in the setting other, as they spent more time close to their home within the housing estates [63]. Furthermore, a recent review summarized that lack of perceived neighbourhood safety may be associated with lower levels of active transport [64]. Given that residents living in a low-SES area often perceive their neighbourhood as less safe compared to high-SES residents [65], safety concerns could be another reason for the low use of streets by low-SES children. A population survey conducted by the Office for City and Neighbourhood Development of the City of Zurich actually could show that the residents of the low-SES district felt less safe walking alone in their neighbourhood at night [66].

While the differences within the settings park, sport, street and other can be referred to a different frequency of use, the significant differences at other schools and outside remained even after accounting for the total time spent in these settings. As a result, these differences could hardly be explained by a more frequent use or by different dwell times, but rather by actually different behaviour when visiting these settings. It can be hypothesized that children from the high-SES district use other schools predominantly for organized PA, as Swiss sports club often use the school infrastructure for their training, or take part in family-based PA in these settings [19]. In contrast, the low-SES children spend time within this setting in more sedentary activities attending the public childcare services of the City of Zurich during lunchtime or after school. Moreover, Lamprecht and colleagues [52] reported that, firstly, girls are less likely to be a member of a sports club and that this aspect is particularly true for girls with an immigrant background. We were able to confirm this effect in the present study with only 33 % of the low-SES girls being a member of a sports club compared to 44 % of the high-SES girls, which were more often of Swiss nationality. Secondly, the same study found that gymnastic clubs, which often use the school infrastructure for their training, are the most popular sports clubs of Swiss girls [52]. Therefore, we can conclude that the more active use of other schools by the high-SES children can partly be attributed to the fact that the high-SES group contained more girls and those were more likely to be a member of a sports club that uses the school infrastructure for training.

The higher and more active use of the area outside the city of Zurich can also be explained by a higher engagement in structured activities within sports clubs as well as by higher logistical and financial parental support [19, 52, 63]. A previous study reported that children with a high-SES background engaged in family-based PA more often than children from a low-SES background [19]. It can be suggested that high-SES families more frequently left the city than low-SES families to be physically active in commercial PA facilities outside the city.

### Limitations

Our study has several limitations. Although objective accelerometer-based measurements of PA are considered to be valid and reliable, they are associated with different known problems. These include the inability to accurately assess certain activities such as upper body movement or cycling [67], the choice of different processing methods and threshold values to determine MVPA, which can have an impact on the recorded level of PA [68], and reactivity issues reported by Dösegger and colleagues [69]. Inaccurate and missing GPS positions due to poor satellite signal may lead to misclassification errors [48] and, therefore, to under- or overrepresentations of certain activity settings. Despite taking steps to reduce issues with spatial inaccuracy by identifying invalid GPS points and choosing a buffer zone of ten meters around polygons, misclassification bias remains possible. In particular, the use of buffer zones accounting for the measurement error of the GPS devices might have generated new misclassifications, especially affecting the street setting. Moreover, we did not impute missing GPS data. Due to the fact that we chose second graders from one town and only surveyed them during summer months, our results may not be generalizable to other age groups and living contexts, and are not representative for the entire year. In addition to the assessed parameters such as individual socioeconomic factors or weather conditions, also parent’s and children’s subjective perception, their safety concerns and fears can have a crucial influence on children’s independent mobility and activity behaviour. However, as these parameters haven’t been assessed in this study, they were not available for analysis.

## Conclusions

Independent of individual SES, we could observe several differences in the spatial PA and SB pattern between children from a low- and high-SES neighbourhood, which imply a location-specific influence of neighbourhood SES on children’s spatial activity behaviour. Therefore, it is essential for future studies investigating the association between neighbourhood SES and PA or SB not only to use total PA or SB as outcome measure, but to focus more on specific settings in which these behaviours take place. The observed findings further highlight the importance of providing safe street environments and access to appropriate and low-cost recreational facilities to reach recommended PA levels. Furthermore, policies are needed to reduce SB within the home and school setting among children from low- and high-SES neighbourhoods. Further research is required to confirm our findings within different seasons and including children of different ages from different geographic areas. Future studies should also investigate how the subjective perception of the environment can influence the spatial PA and SB pattern of primary school children. Finally, future work should target on methodological aspects in order to improve the accuracy and comparability of studies using combined accelerometer and GPS approaches.

## Additional files

Additional file 1: Multilevel analysis. Statistical models for the four outcome variables. (PDF 71 kb) Additional file 2: STROBE Statement. Checklist of items that should be included in reports of cross-sectional studies. (PDF 107 kb)
