# Supplementary material for: Spatial physical activity patterns among primary school children living in neighbourhoods of varying socioeconomic status: a cross-sectional study using accelerometry and Global Positioning System
Source: BMC Public Health. 2016 Mar 22;16:282. doi: 10.1186/s12889-016-2954-8 (PMC4802637; doi:10.1186/s12889-016-2954-8)
Supplement: Additional file 1: — Multilevel analysis. Statistical models for the four outcome variables. (PDF 71 kb) [file 12889_2016_2954_MOESM1_ESM.pdf]

Multilevel analysis

Model 1: Amount of MVPA (in minutes)

Random effects:

	Chi.sq	Chi.DF	elim.num	p.value
class	0.10	1	1	0.749
id	17.57	1	kept	0.000

Fixed effects:

	Sum Sq	Mean Sq	NumDF	DenDF	F.value	elim.num	p.value
income	2.80	0.56	5	49.20	0.40	1	0.844
education	0.91	0.23	4	50.98	0.16	2	0.956
nationality	3.07	1.02	3	55.86	0.74	3	0.534
membership	0.41	0.41	1	58.96	0.29	4	0.590
bmi	2.20	2.20	1	59.64	1.59	5	0.212
language	3.40	1.70	2	61.53	1.23	6	0.300
week day	12.04	2.01	6	2240.84	1.45	7	0.192
location	786.24	112.32	7	2261.51	81.05	kept	<0.001
sex	28.06	28.06	1	63.72	20.25	kept	0.000
wear time	17.81	17.81	1	70.79	12.85	kept	0.001
district	54.48	54.48	1	88.58	39.31	kept	<0.001
temperature	8.42	8.42	1	62.80	6.08	kept	0.016
precipitation	9.56	9.56	1	62.46	6.90	kept	0.011
location:kreis	136.99	19.57	7	2259.99	14.12	kept	<0.001

Final model:

log(minutes of MVPA) ~ location + sex + wear time + district + temperature + precipitation + (1 | id) + location:district

Model 2: Proportion of time in MVPA (in %)

Random effects:

	Chi.sq	Chi.DF	elim.num	p.value
class	0.19	1	1	0.667
id	45.99	1	kept	<0.001

Fixed effects:

	Sum Sq	Mean Sq	NumDF	DenDF	F.value	elim.num	p.value
education	0.39	0.10	4	48.85	0.16	1	0.955
membership	0.02	0.02	1	53.82	0.03	2	0.858
nationality	0.64	0.21	3	54.46	0.36	3	0.779
income	2.22	0.44	5	59.36	0.76	4	0.585
bmi	0.89	0.89	1	63.11	1.51	5	0.224
language	2.92	1.46	2	63.76	2.49	6	0.091
temperature	2.20	2.20	1	65.35	3.74	7	0.057
precipitation	2.00	2.00	1	67.04	3.40	8	0.070
week day	11.52	1.92	6	2572.97	3.32	kept	0.003
location	315.58	45.08	7	2585.97	77.88	kept	<0.001
sex	22.85	22.85	1	79.50	39.48	kept	<0.001
district	6.19	6.19	1	111.52	10.69	kept	0.001
location:district	32.48	4.64	7	2585.76	8.02	kept	<0.001

Final model:

log(proportion of MVPA) ~ week day + location + sex + district + (1 | id) + location:district

Model 3: Amount of SB (in minutes)

Random effects:

	Chi.sq	Chi.DF	elim.num	p.value
class	1.12	1	1	0.291
id	7.75	1	kept	0.005

Fixed effects:

	Sum Sq	Mean Sq	NumDF	DenDF	F.value	elim.num	p.value
education	1.15	0.29	4	46.50	0.21	1	0.931
membership	0.03	0.03	1	49.21	0.02	2	0.888
nationality	1.95	0.65	3	50.28	0.48	3	0.697
precipitation	0.26	0.26	1	53.71	0.19	4	0.665
bmi	0.61	0.61	1	51.48	0.45	5	0.505
language	4.39	2.20	2	53.70	1.63	6	0.206
week day	44.96	7.49	6	2294.51	5.55	kept	0.000
location	1834.35	262.05	7	2309.31	193.91	kept	<0.001
sex	7.20	7.20	1	57.17	5.33	kept	0.025
wear time	42.60	42.60	1	63.45	31.52	kept	0.000
district	9.42	9.42	1	89.77	6.97	kept	0.010
income	21.59	4.32	5	61.73	3.20	kept	0.013
temperature	5.75	5.75	1	62.26	4.25	kept	0.043
location:district	61.11	8.73	7	2306.73	6.46	kept	0.000

Final model:

log(minutes of SB) ~ week day + location + sex + wear time + district + income + temperature + (1 | id) + location:district

Model 4: Proportion of time in SB (in %)

Random effects:

	Chi.sq	Chi.DF	elim.num	p.value
class	0.00	1	1	1.000
id	55.71	1	kept	<0.001

Fixed effects:

	Sum Sq	Mean Sq	NumDF	DenDF	F.value	elim.num	p.value
nationality	172.55	57.52	3	48.05	0.22	1	0.886
temperature	54.99	54.99	1	51.98	0.21	2	0.652
education	983.04	245.76	4	52.33	0.92	3	0.460
membership	258.99	258.99	1	57.57	0.97	4	0.328
language	609.40	304.70	2	57.29	1.14	5	0.326
bmi	195.40	195.40	1	59.50	0.73	6	0.395
income	2020.03	404.01	5	63.10	1.52	7	0.198
week day	5796.34	966.06	6	2285.91	3.63	kept	0.001
location	82605.12	11800.73	7	2299.27	44.28	kept	<1e-07
sex	1773.43	1773.43	1	66.13	6.66	kept	0.012
district	770.97	770.97	1	82.23	2.89	kept	0.093
precipitation	1269.20	1269.20	1	66.33	4.76	kept	0.033
location:district	12023.93	1717.70	7	2298.87	6.45	kept	0.000

Final model:

log(proportion of SB) ~ week day + location + sex + district + precipitation + (1 | id) + location:district
