# Supplementary material for: Spatial physical activity patterns among primary school children living in neighbourhoods of varying socioeconomic status: a cross-sectional study using accelerometry and Global Positioning System
Source: BMC Public Health. 2016 Mar 22;16:282. doi: 10.1186/s12889-016-2954-8 (PMC4802637; doi:10.1186/s12889-016-2954-8)
Supplement: Additional file 2: — STROBE Statement. Checklist of items that should be included in reports of cross-sectional studies. (PDF 107 kb) [file 12889_2016_2954_MOESM2_ESM.pdf]

STROBE Statement
Checklist of items that should be included in reports of ***cross-sectional studies***

	Item No	Recommendation	Page in manuscript (subsection)
**Title and abstract**	1	(*a*) Indicate the study's design with a commonly used term in the title or the abstract	1 (title)
		(*b*) Provide in the abstract an informative and balanced summary of what was done and what was found	2-3 (abstract)
**Introduction**			
Background/ rationale	2	Explain the scientific background and rationale for the investigation being reported	3-6 (background)
Objectives	3	State specific objectives, including any prespecified hypotheses	6 (last paragraph of background)
**Methods**			
Study design	4	Present key elements of study design early in the paper	7 (setting; participant recruitment)
Setting	5	Describe the setting, locations, and relevant dates, including periods of recruitment, exposure, follow-up, and data collection	7-8 (setting; participant recruitment)
Participants	6a	Give the eligibility criteria, and the sources and methods of selection of participants	7 (participant recruitment)
Variables	7	Clearly define all outcomes, exposures, predictors, potential confounders, and effect modifiers. Give diagnostic criteria, if applicable	8-9 (data collection and measures); 11-12 (data analysis)
Data sources/ measurement	8	For each variable of interest, give sources of data and details of methods of assessment (measurement). Describe comparability of assessment methods if there is more than one group	8-9 (data collection and measures)
Bias	9	Describe any efforts to address potential sources of bias	9-12 (data processing; activity settings; data analyses; 19-20 (limitations)
Study size	10	Explain how the study size was arrived at	7 (setting)
Quantitative variables	11	Explain how quantitative variables were handled in the analyses. If applicable, describe which groupings were chosen and why	9-10 (data processing; activity settings)
Statistical methods	12	(*a*) Describe all statistical methods, including those used to control for confounding	11-12 (data analysis) Additional File 1
		(*b*) Describe any methods used to examine subgroups and interactions	11-12 (data analysis)
		(*c*) Explain how missing data were addressed	11-12 (data analysis); 19-20 (limitations)
		(*d*) If applicable, describe analytical methods taking account of sampling strategy	Not applicable
		(*e*) Describe any sensitivity analyses	Not applicable

Results			
Participants	13	(a) Report numbers of individuals at each stage of study—eg numbers potentially eligible, examined for eligibility, confirmed eligible, included in the study, completing follow-up, and analysed	12-13 (general and socioeconomic characteristics)
		(b) Give reasons for non-participation at each stage	12-13 (general and socioeconomic characteristics)
		(c) Consider use of a flow diagram	Not applicable
Descriptive data	14	(a) Give characteristics of study participants (eg demographic, clinical, social) and information on exposures and potential confounders	12-13 (general and socioeconomic characteristics);<br>Table 1
		(b) Indicate number of participants with missing data for each variable of interest	Table 1-3
Outcome data	15	Report numbers of outcome events or summary measures	12-14 (results)<br>Table 2-3
Main results	16	(*a*) Give unadjusted estimates and, if applicable, confounder-adjusted estimates and their precision (eg, 95% confidence interval). Make clear which confounders were adjusted for and why they were included	Not applicable
		(*b*) Report category boundaries when continuous variables were categorized	Not applicable
		(*c*) If relevant, consider translating estimates of relative risk into absolute risk for a meaningful time period	Not applicable
Other analyses	17	Report other analyses done—eg analyses of subgroups and interactions, and sensitivity analyses	Not applicable
**Discussion**			
Key results	18	Summarise key results with reference to study objectives	14-15 (first paragraph of discussion)
Limitations	19	Discuss limitations of the study, taking into account sources of potential bias or imprecision. Discuss both direction and magnitude of any potential bias	19-20 (limitations)
Interpretation	20	Give a cautious overall interpretation of results considering objectives, limitations, multiplicity of analyses, results from similar studies, and other relevant evidence	14-21 (discussion; limitations; conclusion)
Generalisability	21	Discuss the generalisability (external validity) of the study results	19-20 (limitations)
**Other information**			
Funding	22	Give the source of funding and the role of the funders for the present study and, if applicable, for the original study on which the present article is based	21 (competing interests)

Note: An Explanation and Elaboration article discusses each checklist item and gives methodological background and published examples of transparent reporting. The STROBE checklist is best used in conjunction with this article (freely available on the Web sites of PLoS Medicine at http://www.plosmedicine.org/, Annals of Internal Medicine at http://www.annals.org/, and Epidemiology at http://www.epidem.com/). Information on the STROBE Initiative is available at www.strobe-statement.org.
